# 57 HIV Infection Increases Risk of Sepsis and Other Complications in Burn Patients

**DOI:** 10.1093/jbcr/irae036.049

**Published:** 2024-04-17

**Authors:** Dalton Amador, Isabel B Obias, Carolina Segura, Yash Ramgopal, Chris K Soudah, Juquan Song, Amina E I Ayadi, Georgiy Golovko, Steven E Wolf

**Affiliations:** University of Texas Medical Branch, Houston, TX; University of Texas Medical Branch, Galveston, TX; University of Texas Medical Branch at Galveston, Galveston, TX; University of Texas Medical Branch, Blocker Burn Unit, Galveston, TX; University of Texas Medical Branch, Houston, TX; University of Texas Medical Branch, Galveston, TX; University of Texas Medical Branch at Galveston, Galveston, TX; University of Texas Medical Branch, Blocker Burn Unit, Galveston, TX; University of Texas Medical Branch, Houston, TX; University of Texas Medical Branch, Galveston, TX; University of Texas Medical Branch at Galveston, Galveston, TX; University of Texas Medical Branch, Blocker Burn Unit, Galveston, TX; University of Texas Medical Branch, Houston, TX; University of Texas Medical Branch, Galveston, TX; University of Texas Medical Branch at Galveston, Galveston, TX; University of Texas Medical Branch, Blocker Burn Unit, Galveston, TX; University of Texas Medical Branch, Houston, TX; University of Texas Medical Branch, Galveston, TX; University of Texas Medical Branch at Galveston, Galveston, TX; University of Texas Medical Branch, Blocker Burn Unit, Galveston, TX; University of Texas Medical Branch, Houston, TX; University of Texas Medical Branch, Galveston, TX; University of Texas Medical Branch at Galveston, Galveston, TX; University of Texas Medical Branch, Blocker Burn Unit, Galveston, TX; University of Texas Medical Branch, Houston, TX; University of Texas Medical Branch, Galveston, TX; University of Texas Medical Branch at Galveston, Galveston, TX; University of Texas Medical Branch, Blocker Burn Unit, Galveston, TX; University of Texas Medical Branch, Houston, TX; University of Texas Medical Branch, Galveston, TX; University of Texas Medical Branch at Galveston, Galveston, TX; University of Texas Medical Branch, Blocker Burn Unit, Galveston, TX; University of Texas Medical Branch, Houston, TX; University of Texas Medical Branch, Galveston, TX; University of Texas Medical Branch at Galveston, Galveston, TX; University of Texas Medical Branch, Blocker Burn Unit, Galveston, TX

## Abstract

**Introduction:**

Infection with human immunodeficiency virus (HIV) confers an immunocompromised status that renders patients susceptible to various pathogen-related morbidities, eventually progressing to acquired immunodeficiency syndrome (AIDS) absent antiretroviral therapy. Burn patients are also susceptible to infection due to trauma-related tissue breakdown and organ dysfunction impairing host defense and providing avenues for pathogen entry and proliferation. The purpose of this study is to explore the respective relative risks of pathogen-related complications in burn patients with HIV infection compared to those without.

**Methods:**

This study utilized a research database that provides real-time access to de-identified medical records. Burn patients were identified using the relevant ICD-10 codes and were stratified between those who had and had not received a previous diagnosis of HIV. Subsequently risk ratios and differences between the two groups for sepsis, pneumonia, myocardial infarction, respiratory failure, acute kidney injury, and death occurring within three months of injury were generated and analyzed using the database.

**Results:**

664,357 burn patients from 59 healthcare organizations were identified using the database. These patients were then sorted according to infection with HIV (N = 2,437) and without infection with HIV (N = 661,920) cohort. The two cohorts were balanced using (1:1) propensity score matching for age, sex, race, ethnicity, and total burn surface area (TBSA).

Burn patients with HiV infection demonstrated a greater risk of developing sepsis (risk ratio [RR], 2.800; 95% confidence interval, [1.686, 4.650]; p < 0.001), pneumonia (RR, 3.000; CI, [1.897, 4.745]; p < 0.001), myocardial infarction (RR, 2.167; CI, [1.096, 4.284]; p = 0.023), respiratory failure (RR, 1.535; CI, [1.050, 2.244]; p = 0.026), acute kidney injury (RR, 2.319; CI, [1.655, 3.249]; p < 0.001), and mortality (RR, 2.000; CI, [1.139, 3.512] p = 0.014) within three months of injury.

**Conclusions:**

HIV infection was associated with a greater risk of sepsis, pneumonia, myocardial infarction, respiratory failure, acute kidney injury, and death in burn patients within three months of injury.

**Applicability of Research to Practice:**

HIV infection in burn patients may be regarded as a risk factor for the various complications and morbidities analyzed in this study and guide anticipatory clinical decision-making. Future studies may explore these associations more.

